# Urinary exosomal activating transcriptional factor 3 as the early diagnostic biomarker for sepsis-induced acute kidney injury

**DOI:** 10.1186/s12882-016-0415-3

**Published:** 2017-01-07

**Authors:** Tanaporn Panich, Wiwat Chancharoenthana, Poorichaya Somparn, Jiraphorn Issara-Amphorn, Nattiya Hirankarn, Asada Leelahavanichkul

**Affiliations:** 1 Immunology Unit, Department of Microbiology, Chulalongkorn University, Bangkok, 10330 Thailand; 2 Division of Nephrology, Department of Medicine, Chulalongkorn University, Bangkok, 10330 Thailand; 3 Research Affairs, Faculty of Medicine, Chulalongkorn University, Bangkok, 10330 Thailand; 4 Division of Nephrology and Hypertension, Department of Medicine, Princess Chulabhorn College of Medical Sciences, Chulabhorn Royal Academy of Science (CRAS), Bangkok, 10210 Thailand; 5 Center of Excellence in Immunology and Immune-mediated Diseases, Department of Microbiology, Chulalongkorn University, Bangkok, 10330 Thailand; 6 Medical Microbiology, Interdisciplinary Program, Graduate School, Chulalongkorn University, Bangkok, Thailand

**Keywords:** Activating transcriptional factor 3, Acute kidney injury, Biomarker, Neutrophil gelatinase-associated lipocalin, Urine exosome

## Abstract

**Background:**

An early sepsis-induced acute kidney injury (sepsis-AKI) biomarker is currently in needed. Urinary neutrophil gelatinase-associated lipocalin (uNGAL) is a candidate of sepsis-AKI biomarker but with different cut-point values. Urinary exosomal activating transcriptional factor 3 (uATF3) has been mentioned as an interesting biomarker.

**Methods:**

We conducted experiments in mice and a prospective, multicenter study in patients as a proof of concept that urine exosome is an interesting biomarker. An early expression of ATF3 in kidney of CD-1 mice at 6 h after cecal ligation and puncture implied the possibility of uATF3 as an early sepsis-AKI biomarker. Increase serum creatinine (Scr) ≥0.3 mg/dL from the baseline was used as an AKI diagnosis and urine was analyzed for uATF3 and uNGAL. Patients with baseline Scr at admission ≥1.5 mg/dL were excluded.

**Results:**

The analysis showed higher Scr, uNGAL and uATF3 in patients with sepsis-AKI in comparison with patients with sepsis-non-AKI and healthy volunteers. A fair correlation, r^2^ = 0.47, between uATF3 and uNGAL was showed in sepsis-AKI group with Scr ≥2 mg/dL. To see if uATF3 could be an early sepsis-AKI biomarker, urine sample was collected daily during the first week of the admission. In sepsis-AKI and sepsis-non-AKI groups, uNGAL were 367 ± 43 ng/mL and 183 ± 23 ng/mL, respectively; and uATF3 were 19 ± 4 ng/mL and 1.4 ± 0.8 ng/mL, respectively. With the mean value of uNGAL and uATF3 in sepsis AKI as a cut-off level, AUROC of uNGAL and uATF3 were 64% (95% CI 0.54 to 0.74) and 84% (95% CI 0.77 to 0.91), respectively.

**Conclusions:**

Urine exosome is an interesting source of urine biomarker and uATF3 is an interesting sepsis-AKI biomarker.

## Background

Sepsis is the systemic inflammatory response syndrome (SIRS) in the response of infectious processes regardless of organisms [1]. Sepsis is a world-wide health care problem and a leading cause of death of patients in intensive care unit [2]. On the other hand, acute kidney injury (AKI) is a clinical syndrome of an abrupt renal dysfunction leading to uremic toxins accumulation and several organs dysfunction [3]. It is interesting that more than 50% of patients with sepsis develop AKI and sepsis patients with AKI had a higher mortality rate [4, 5]. The definition of sepsis-induced AKI (sepsis-AKI) is an AKI occurs simultaneously or subsequently after sepsis with the exclusion of other causes of AKI [2]. Despite an advance in an understanding of pathogenesis and sepsis-AKI therapy in animal models, the translational research into a human condition is currently unsuccessful. This, at least in part, demonstrates the requirement for early sepsis-AKI biomarkers [6]. Serum creatinine (Scr), a current standard biomarker of AKI, is a late renal injury biomarker which does not a good representative of sepsis-AKI [7–10]. Thus, several potential biomarkers for detecting sepsis-AKI have been studies. Urinary neutrophil gelatinase associated lipocalin (uNGAL) is one of the promising sepsis-AKI biomarker, but still have a debate on the cut-off value [11, 12]. The difference cut-off value of uNGAL might due to the possibility of different sources of neutrophil gelatinase-associated lipocalin (NGAL), such as neutrophil and other internal organs, etc., especially, in patients with sepsis [13, 14].

Exosomes are the nano-vesicles released by most of the cells as a method of cell communication for either normal physiologies or many pathological processes [15]. These nano-vesicles are some parts of cell membrane containing several proteins including the genetic materials [16, 17]. In another word, exosome is a membrane-protected source of biological materials. Hence, urine exosome is an interesting source of urine biomarkers [17–19]. Activating transcription factor 3 (ATF3) is one of the transcriptional factor protected inside urine exosome which expressed in urine of AKI in patients and animal models [20, 21]. Renal ATF3 is activated in mouse models of ischemic reperfusion injury and cisplatin induced nephrotoxicity [22, 23]. Interestingly, ATF3 downstream functions are anti-apoptosis and anti-inflammation through the attenuation of pro-inflammatory mediators and the alteration of the chromatin binding site in transcriptional factors [21, 22, 24, 25]. Hence, ATF3 should be involved in the pathophysiology of sepsis, AKI and sepsis-AKI. Unfortunately, ATF3 detection in total urine and non-exosomal urine fraction is inconsistent [20, 21] due, in part, to the degradation of transcriptional factor in urine [26]. But ATF3 seems to be protected inside exosome membrane and allow easier detection [20]. Therefore, we hypothesized that urinary exosomal ATF3 (uATF3) should be an interesting biomarker of sepsis-AKI.

## Materials and methods

### Cecal ligation and puncture sepsis model and kidney immunohistochemistry

The US National Institutes of Health (NIH) criteria and protocols were followed. Male, 8–10 week old CD-1 mice (National Laboratory Animal Center, Nakhon Pathom, Thailand) were used. Cecal ligation and puncture sepsis model (CLP) was performed under isoflurane anesthesia as previously details [10, 27]. In brief, cecum was ligated at 12 mm from the cecal tip, punctured with a 21-gauge needle and squeezed for a small amount of feces. Supplementary fluid, 1 mL of normal saline, was administered at the post-operational period. However, antibiotic was not used because the effect of antibiotic to urinary exosome was uncertain. Only the abdominal incision for cecal identification was done in sham group. Blood (50 μL) was collected through tail vein nicking for the time-point analysis of mouse serum NGAL (sNGAL) (R&D systems, MN, USA) and serum creatinine (Scr) (QuantiChrom Creatinine assay DICT-500, Hayward, CA, USA). Mice were euthanized under isoflurane anesthesia. Kidney, liver and spleen were put in liquid nitrogen then kept at −80 °C until used. Kidney was also fixed in 10% neutral buffered formalin and embedded in paraffin for the immunohistochemical section of 45 μm thickness with anti-mouse NGAL (R&D systems, MN, USA) and anti-mouse ATF3 (Santa cruz, CA, USA) as previously described [27]. Western Blot analysis of tissue ATF3 and activated caspase-3 (Cell Signaling, MA, USA) were also performed. The protocol for the experiment was approved by the Animal Experimentation Ethics Committee of Chulalongkorn University.

### Patient selections

Spot urine collection was performed during January 2014 to August 2014 at the King Chulalongkorn Memorial Hospital (KCMH) and three other general hospitals. To include patients with documented early sepsis-AKI during the hospitalization, all adult patients (age more than 18-year-old) in internal medicine ward with sepsis by the demonstration of systemic inflammatory response syndrome (SIRS) with the evidence or the suspicious of infectious causes [1]. Additionally, AKI defined as Scr increase ≥0.3 mg/dL from baseline followed KDIGO and AKIN criteria [3]. Then sepsis-induced AKI (sepsis-AKI) defined as sepsis with Scr increase ≥0.3 mg/dL from baseline [3]. Otherwise, patients with sepsis but Scr increase ≤0.3 mg/dL were classified as sepsis-non-AKI. Exclusion criteria were (*i*) patients with the baseline Scr (first Scr measured) higher than 1.5 mg/dL or other history of chronic kidney diseases, (*ii*) pregnant women, (*iii*) chronic systemic diseases with proteinuria such as diabetic nephropathy, lupus nephritis, etc.; (*iv*) obstructive uropathy, and (*v*) post kidney transplantation. Baseline Scr were retrospectively reviewed from the medical records of at least 3-month earlier from the participant hospitals. Patients with sepsis with elevated Scr at the time of admission was firstly determined as sepsis induced acute kidney injury unless they met the exclusion criteria (1^st^ time Scr >1.5 mg/dL). Renal sonogram was used to exclude the preexisting chronic kidney disease (CKD) in some patients. Then the participants were followed with daily first morning spot urine and serum samples collection during the 1^st^ week of admission period.

**Fig. 1:**
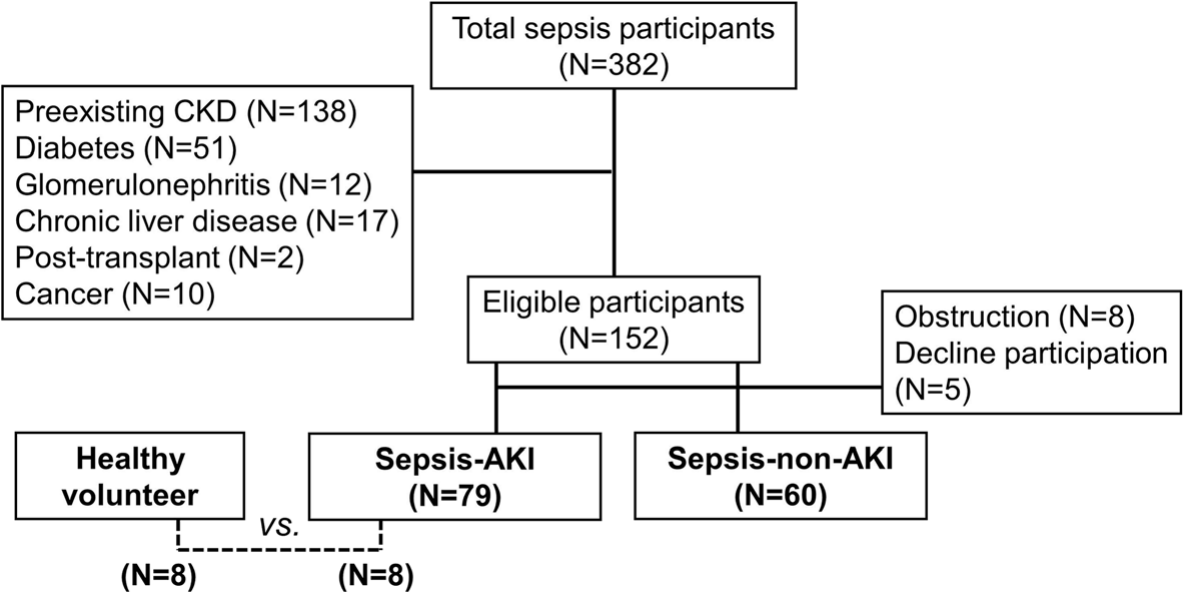
Consort-type flowchart showing patient selection

Of all 382 patients, most of the patients had underlying diseases and only 139 patients were included and approximately half of these patients were sepsis-AKI (*n* = 79) (Fig. 1 and Table 1) implied the importance of AKI in sepsis. Then 8 patients with sepsis-AKI were randomly selected by propensity score matched in gender, age and comorbidity to compare with 8 healthy volunteers group served as discovery set. Subsequently, all urine samples from patients who underwent the new onset of sepsis-AKI (*n* = 79) during the 1^st^ week of admission were analyzed in comparison with sepsis-non-AKI (*n* = 60). Of note, there were 17 and 11 patients of sepsis-AKI and non sepsis-AKI, respectively, transferred to intensive care unit. For the urine collection, urine was centrifuged at 1,000x *g* for 10 min to remove debris, and stored at −80 °C until used.

**Table 1:** Patient characteristics

Characteristics	Healthy volunteers (*n* = 8)	Sepsis-AKI (*n* = 79)	Sepsis-non-AKI (*n* = 60)	*p*-value
Mean age, yr (SD)	43.1 (10.2)	63.3 (13.3)	51.5 (18.5)	<0.0001
Men (%)	3 (37.5)	52 (65.8)	28 (46.7)	0.04
Baseline Scr (mg/dL)	0.9 ± 0.1	0.9 ± 0.1	0.9 ± 0.3	0.10
All-time point Scr (mg/dL)				
Median (P_25_-P_75_)	0.9 (0.8-1.0)	3.2 (1.7-4.3)	1.0 (0.8-1.3)	<0.0001
All-time point MDRD Scr-eGFR				
(mL/min/1.73 m^2^)	109.3	33.5	64.8	<0.0001
Median (P_25_-P_75_)	(92.1-128.3)	(19.8-42.2)	(48.2-70.2)	
APACHE II score (SD)	-	22 (1.9)	18 (6.4)	<0.0001
Principle comorbidities (%)				
Hypertension	-	26 (32.9)	22 (36.7)	0.77
Cardiovascular disease	-	10 (12.7)	6 (10.0)	0.82
COPD	-	7 (8.9)	1 (1.7)	0.15
No known underlying disease	-	26 (32.9)	18 (30.0)	0.86
Causes of sepsis				
Pneumonia	-	36 (45.6)	22 (36.7)	0.38
Urinary tract infection	-	28 (35.4)	17 (28.3)	0.48
Intra-abdominal infection	-	7 (8.9)	12 (20.0)	0.10
Soft tissue infection	-	0 (0)	7 (11.7)	0.006
Tropical infection	-	8 (10.1)	2 (3.3)	0.23
Outcomes				
Severe sepsis (%)	-	43 (54.4)	18 (30.0)	0.007
Intensive care unit admission (%)	-	31 (39.2)	13 (21.7)	0.04
In-hospital length of stay, days (SD)	-	63 (15.6)	30 (11.9)	<0.0001
Dialysis (%)	-	25 (31.6)	0 (0)	<0.0001
Mortality (%)	-	9 (11.4)	4 (6.7)	0.52
AKI severity (%)				
AKIN 1	-	38 (48.1)	0	<0.0001
AKIN 2	-	30 (38.0)	0	<0.0001
AKIN 3	-	28 (35.4)	0	<0.0001

*AKI*, acute kidney injury; *AKIN*, Acute Kidney Injury Network; *APACHE II*, Acute Physiology and Chronic Health Evaluation II; *COPD*, chronic obstructive pulmonary disease; *MDRD* Scr-eGFR, GFR estimated from Scr using the Modification of Diet in Renal Disease equation; P25-P75, 25^th^ percentile to 75^th^ percentile; *Scr*, serum creatinine

This study was approved by the Institutional Review Board of Faculty of Medicine, Chulalongkorn University, Bangkok, Thailand and adhered to ARRIVE guideline/methodology.

### Measurement of serum creatinine, urine NGAL and urinary exosomal ATF3 of patients’ sample

uNGAL and Scr were measured by ELISA assay (R&D systems, MN, USA) and enzymatic based automated analyzer, respectively. Urinary exosomal ATF3 was measured by Western blot analysis with the quantitative analysis by the calculation from recombinant ATF3 (Santa cruz, CA, USA). Urine exosomes were isolated by the differential centrifugation method as described previously [20]. In brief, frozen urine was thawed and 10 mL of urine was centrifuged at 17,000x *g* for 15 min. Then the supernatant from the first spin was centrifuged at 200,000x *g* for 1 h and suspended by 50 μL of 1% SDS in 50 mM Tris. The analysis of Tumor susceptible gene 101 (TSG101), one of the exosome biomarkers [28], was used to support the efficiency of this method. The initial urinary exosome-associated proteins (50 μL) were loaded in different volume into each well depended on urine creatinine (Ucr). This method of exosome biomarker normalization by Ucr was also described previously [20, 29]. The quantitative values of ATF3 by Western blot analysis were calculated from the density of 3 known concentrations of recombinant ATF3 (MyBiosource, CA, USA) loaded in every gel along with the samples.

## Western blot analysis

Sample protein were separated by 1day sodium dodecyl sulfate (SDS)–polyacrylamide gel electrophoresis and transferred into a nitrocellulose membrane under Towbin transfer buffer (TBS). The membranes were blocked with 5% milk in TBS plus 0.1% tween-20 for 1 h at room temperature then probed overnight at 4 °C with anti-mouse ATF3 and anti-mouse activated caspase-3 (Cell Signaling, MA, USA) for mouse internal organs or with anti-human ATF3 (Santa cruz, CA, USA) and anti-TSG101 (Cell Signaling, MA, USA) for urine exosome samples. To see if there is TSG101 in the non-exosome urine fraction after centrifuged at 200,000x g (soup), acetone precipitation was used to concentrate the soluble protein in soup fraction before Western blot analysis in parallel with urine exosome fraction (Fig. 4a). For the visualization, the blot was incubated with appropriate horseradish peroxidase-linked secondary antibodies (1:5,000) (Jackson ImmunoResearch Laboratories, PA, USA) and visualized for the chemiluminescence with SuperSignal® (Thermo Scientific, IL, USA) and C-DiGit® Blot Scanner (Li-Cor BioTech, NE, USA).

### Statistical analysis

Data was demonstrated as mean ± SD and the differences between groups were examined for statistical significance by unpaired student *t*-test or one-way analysis of variance (ANOVA) with Tukey’s comparison test in the analysis of experiments with 2 and 3 groups, respectively. Time-point data was examined by two-way ANOVA with Bonferroni post hoc analysis. Survival analysis was performed by log-rank test. *P* value <0.05 was considered to be a statistically significant. Area under receiver operating characteristic (AUROC), sensitivity and specificity were calculated in relative to increased Scr ≥0.3 mg/dL from baseline as the sepsis-AKI diagnosis. The logistic regression model was fitted to the data to determine the influence of uNGAL and uATF3 and also adjusted by those clinical variables (age, comorbidity, APACHE II score) that were shown to have influence on the risk of AKI. The ability of these models to predict AKI was analyzed by the Hosmer–Lemeshow goodness-of-fit test. Then, the discriminative ability of uNGAL and uATF3 for AKI diagnosis was determined by the AUROC. In addition, the net reclassification improvement (NRI) and the category-free NRI (cfNRI) were performed to demonstrate the additive value of preexisting risk prediction model. NRI demonstrates the quality of predicted probabilities as a consequence of adding a new marker to the existing model. Statistical analysis was performed using SPSS for Windows 15.0 (SPSS for Windows; Chicago, IL) and GraphPad Prism (version 6.00; GraphPad Software, La Jolla, CA) software. Reclassification analyses were performed in R using the regression modelling strategies (rms) package for calculating NRI and cfNRI.

## Results

### Both NGAL and ATF3 expressed early in kidney of sepsis mice

**Fig. 2:**
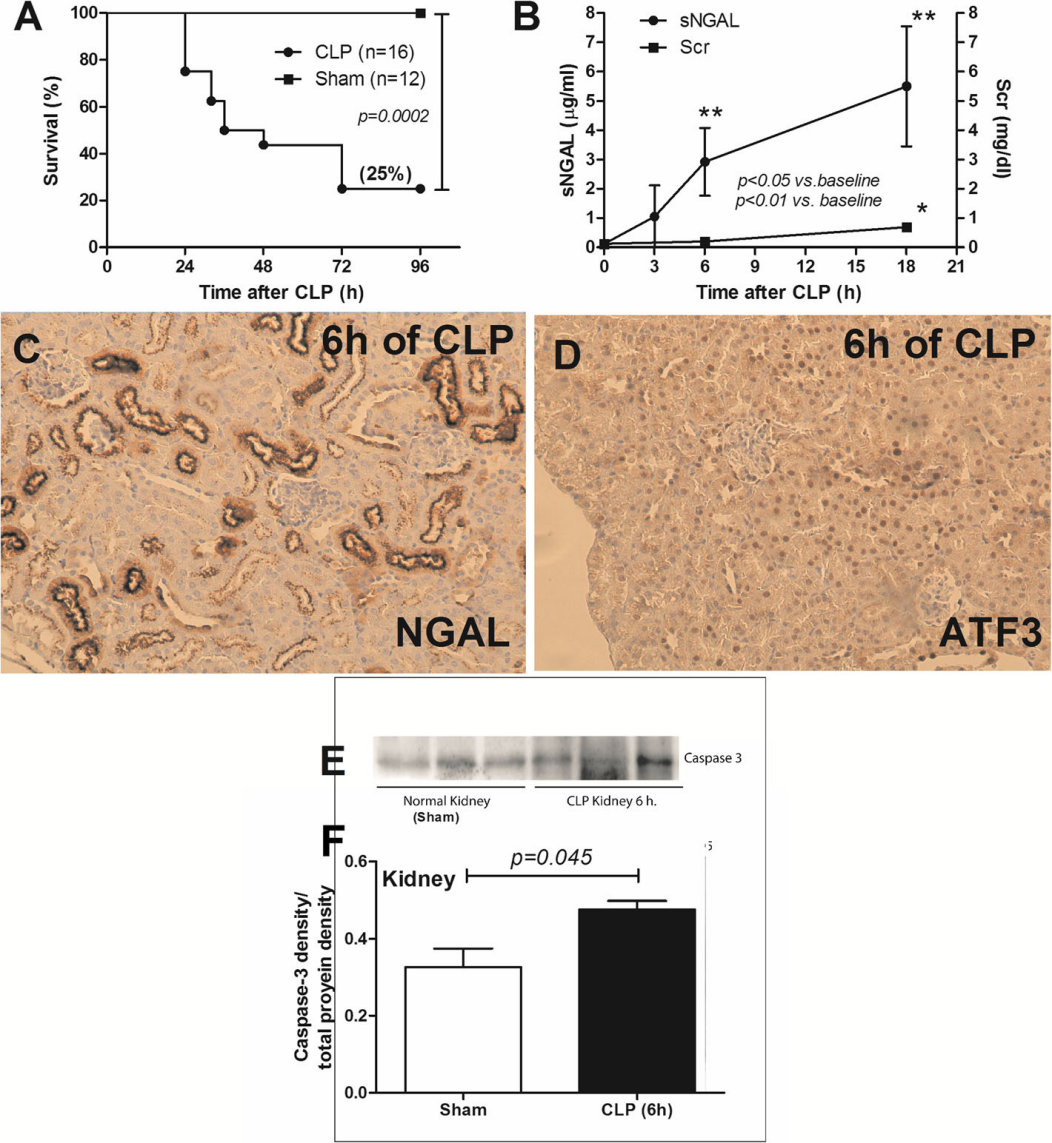
Survival analysis of the cecal ligation and puncture (CLP) model used in the study (**a**) and time-course of serum neutrophil gelatinase associated lipocalin (sNGAL) and serum creatinine (Scr) (**b**) were demonstrated. The representative pictures of the immunohistochemistry of mouse kidney after 6 h of CLP sepsis surgery for NGAL **(c)** and activating transcriptional factor 3 (ATF3) (**d**). NGAL and ATF3 were demonstrated in cytoplasm/brush border and nuclei of cortical renal tubules cells, respectively, and both parameters could not be detected in kidney of sham surgery (data not showed). Additionally, activated caspase-3 was also analyzed in kidney sample at 6 h of CLP and sham surgery (**e**). The comparison of Caspase-3 Western Blot density between kidney of sham and CLP group measured from C-DiGit® Blot Scanner was also showed (**f**)

**Fig. 3:**
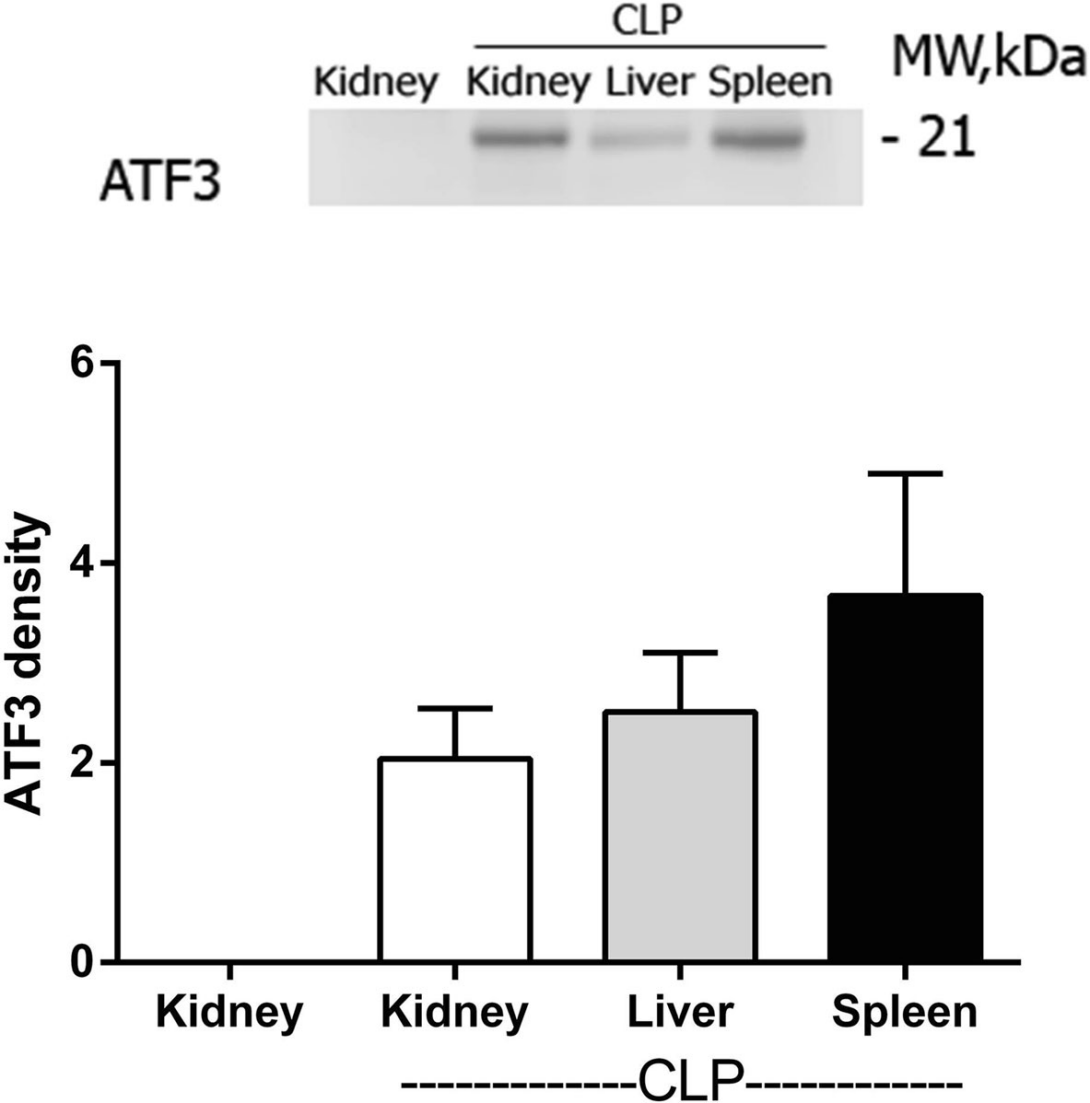
The Western blot analysis of ATF3 in different organs of mice at 6 h after cecal ligation and puncture sepsis surgery (*upper panel*) and the density scoring (l*ower panel*) (*n* = 3-5/group). The reciprocal organs of mice at 6 h after sham surgery were undetectable (data showed only kidney of sham group)

Lethal sepsis model of cecal ligation and puncture (CLP) with survival rate at 25% (Fig. 2a) was used. Although Scr did not significantly increase in the first 6 h after CLP, mice clinical symptoms of illness and serum NGAL (sNGAL) were observed at 6 h of the surgery (Fig. 2b) consistent with previous publications [10, 27]. At 18 h both Scr and sNGAL were higher than the baseline (Fig. 2b). Because at 6 h-post CLP was the earliest time-point of increased sNGAL, kidney histology at 6 h after CLP was analyzed for NGAL and ATF3 as early-sepsis biomarker candidates. Indeed, NGAL expressed in cytoplasm and ATF3 expressed in nuclei of the renal tubular cell (Fig. 2c, d) at 6 h after CLP but not in sham group (data not showed). Moreover, the activated caspase-3 also detectable in kidney at 6 h of CLP by Western blot analysis (Fig. 2e, f). In addition, ATF3 and NGAL expressed in kidney, liver and spleen at 6 h of CLP sepsis mice (Fig. 3) but not in sham (showed only kidney). Due to an early anuria in sepsis mice, we could not analyze urine biomarker in this model.

### High urinary NGAL (uNGAL) and urinary exosomal ATF3 (uATF3) in an initial analysis of patients with sepsis-induced acute kidney injury (Sepsis-AKI)

**Fig. 4:**
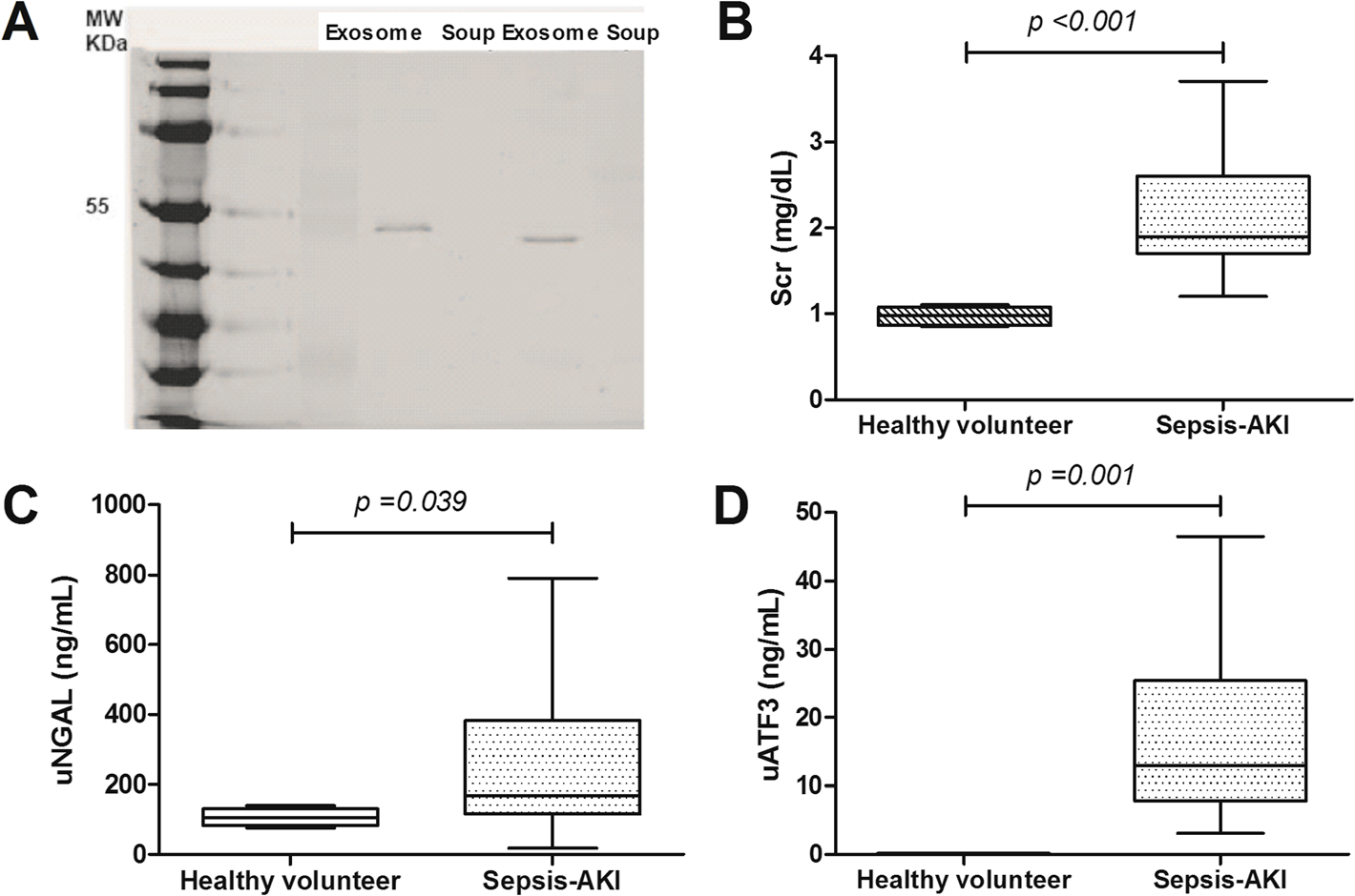
TSG101 (MW 49 kDa) was demonstrated in esosome fraction of urine (Exosome) supported the efficiency of urine exosome extraction procedure and TSG101 could not be detected in non-exosome urine fraction (soup) (**a**). The cross-sectional analysis from sepsis-AKI patients regardless of the onset of sepsis-AKI for serum creatinine (Scr) (**b**), urinary neutrophil gelatinase associated lipocalin (uNGAL) (**c**) and urinary activating transcriptional factor 3 (uATF3) (**d**) were showed (n = 8/group)

The presentation of exosome in urine with extraction procedures was demonstrated by TSG101 (Fig. 4a). By using propensity score matched in gender, age and comorbidity, 8 from 79 patients with sepsis-AKI were randomly selected for the analysis. Scr of patients with sepsis-AKI and healthy volunteers were 2.15 ± 0.22 and 0.97 ± 0.02 mg/dL, respectively (*p* <0.001) (Fig. 4b). With simultaneous analysis, uNGAL and uAFT3 of sepsis-AKI versus healthy volunteers were 1,909 ± 838 versus 17 ± 4 ng/mL and 16 ± 4 and 0 ng/mL, respectively (Fig. 4c, d). Interestingly, uATF3 was non-detectable in all healthy volunteers and both uATF3 and uNGAL associated with AKI severity by AKIN classification. The median value of uATF3 versus uNGAL for AKIN stage 1, 2 and 3 were 8.5-11.7, 14.2-16.7, and 18.2-22.3 ng/mL versus 121–374, 529–871, and 1,071-1,533 ng/mL, respectively (*p* <0.001 among each stage). However, the correlation between all uNGAL and uATF3 was limited (*r* ^2^ = 0.39) but increased (*r* ^2^ = 0.47) with the selection of Scr ≥2 mg/dL. With Scr < 2 mg/dL, there was even lesser correlation (*r* ^2^ = 0.26).

### Urinary NGAL (uNGAL) and urinary exosomal ATF3 (uATF3) in a 7 days follow-up of patients with sepsis-induced acute kidney injury (Sepsis-AKI) showed a potential as an early sepsis-AKI biomarkers

**Fig. 5:**
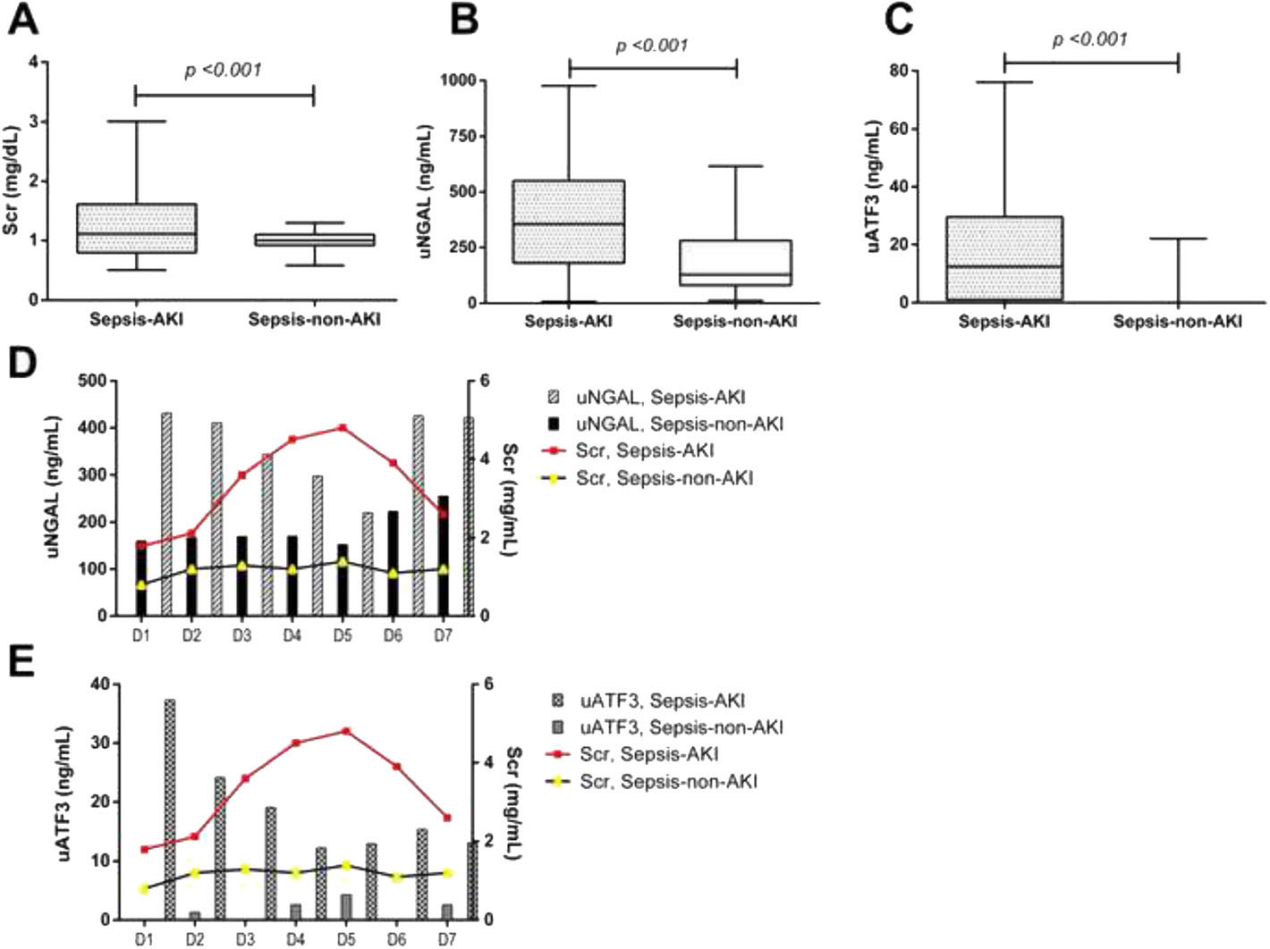
The longitudinal follow-up for 7 days of patients either sepsis-AKI or sepsis-non-AKI (**a**-**c**) with the dose–response relationship between biomarker concentration and severity of AKI, Scr versus uNGAL (**d**) and Scr versus uATF3 (**e**)

There were data from 79 and 60 patients with sepsis-AKI and non sepsis-AKI, respectively, available for the analysis. Of note, 10 from 79 patients (12.7%) in sepsis-AKI group were classified as septic shock. Other patients were categorized as severe sepsis due to there was some organ injuries in these patients. Because there was different onset of sepsis-AKI during 1 week of the follow-up, all data of the 7 days follow-up were combined. With the combination data, Scr, uNGAL and uATF3 of sepsis-AKI and sepsis-non-AKI group were 1.25 ± 0.11 mg/dl, 367 ± 43 ng/mL and 19 ± 4 ng/mL and 0.97 ± 0.02 mg/dL, 183 ± 23 ng/mL and 1.4 ± 0.8 ng/mL, respectively (*p* <0.001 all) (Fig. 5a-c). Of note, most of the patients in this study were non-oligulic sepsis-AKI (68/79 or 86%) (urine output more than 0.5 mL/kg/h) during the observational period. As expected, uNGAL in sepsis-AKI was higher than sepsis non-AKI group reciprocal to previous studies [30]. Interestingly, uATF3 was also higher in sepsis-AKI group. It is interesting that uATF3 was undetectable in nearly all time-points of sepsis non-AKI group implied the high specificity of uATF3 for sepsis-AKI (Fig. 5c). Although both uNGAL and uATF3 correlated with Scr levels, uATF3 provided the better differentiation between sepsis-AKI and sepsis non-AKI (Fig. 5d, e).

**Fig. 6:**
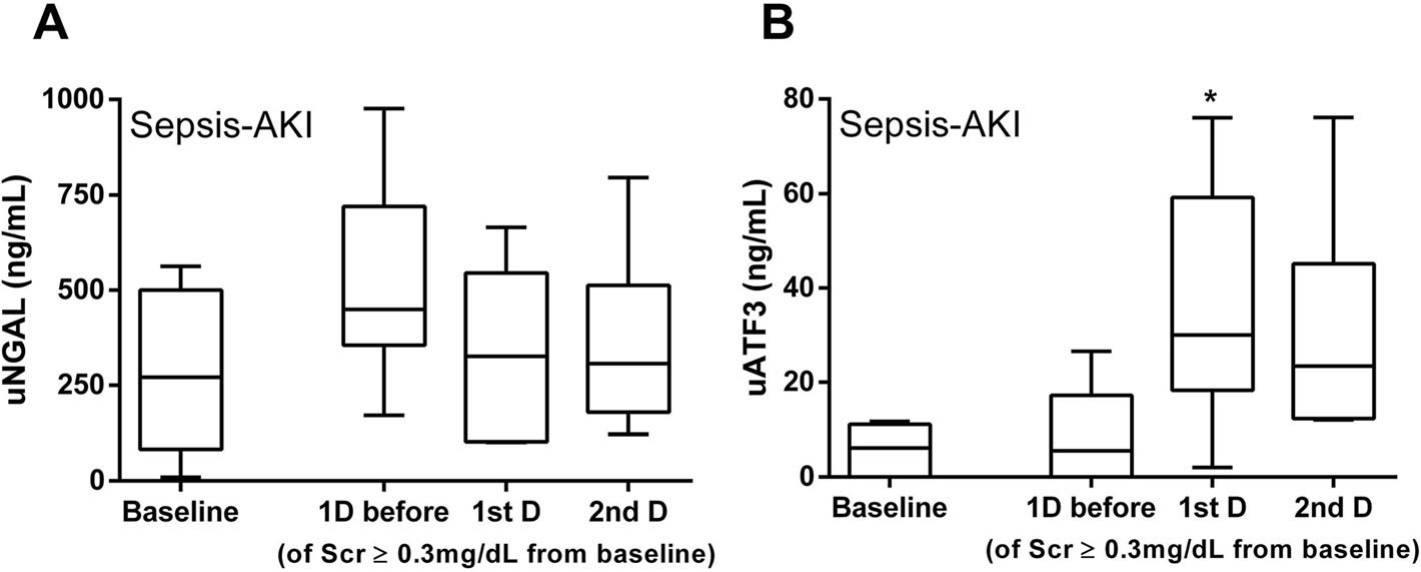
To clearly demonstrated if new biomarkers could be detected at an early stage of sepsis-AKI, values of urinary neutrophil gelatinase associated lipocalin (uNGAL) (**a**) and urinary activating transcriptional factor 3 (uATF3) (**b**) at baseline, 1 day before and at 1^st^ and 2^nd^ day of Scr higher than baseline for 0.3 mg/dL was showed. *p <0.05 compared with baseline levels

**Fig. 7:**
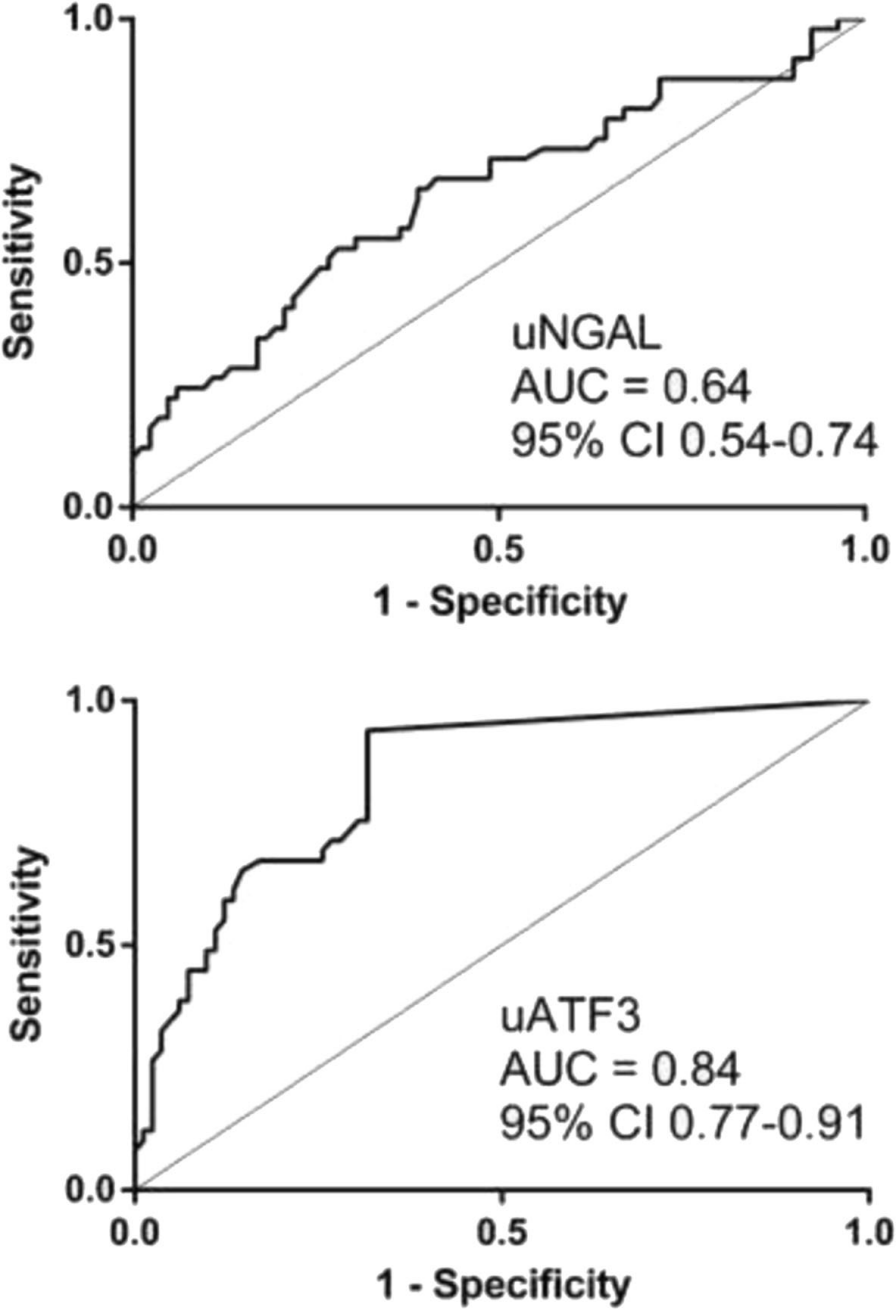
The area under the receiver operating characteristic (AUROC) curve analysis of sensitivity and specificity of the urinary neutrophil gelatinase associated lipocalin (uNGAL) (**a**) and urinary activating transcriptional factor 3 (uATF3) (**b**) with the alteration of Scr higher than 0.3 mg/dL as a gold standard of sepsis-AKI diagnosis

To explore an early sepsis-AKI biomarker potential, uNGAL and uATF3 at 1 day before and at 1^st^ and 2^nd^ day of Scr ≥0.3 mg/dL from baseline was demonstrated (Fig. 5d, e, 6a and b). There was a statistical significant difference of 1^st^ day uATF3 following AKI onset compared with baseline (p <0.05) (Fig. 6b). However, uNGAL but not uATF3 showed a tendency of the higher value at 1 day before AKI onset (Fig. 6a). To calculate area under receiver operating characteristic (AUROC) curve and sensitivity/specificity of uNGAL and uATF3 for sepsis-AKI diagnosis, all time-point data of matched value between uNGAL and uATF3 from patients with sepsis-AKI were combined. The gold standard of sepsis-AKI was Scr ≥0.3 mg/dL from baseline and the cut-off value of uNGAL and uATF3 was 188 and 12 ng/mL, respectively, from the mean level of sepsis-non-AKI group. We found that AUROC of uNGAL and uATF3 were 64% and 84%, respectively (Fig. 7). It seems that uATF3 showed a comparable sensitivity but higher specificity for sepsis-AKI diagnosis in comparison to uNGAL. Urine NGAL and uATF3 performance with different cut-off values were demonstrated (Table 2). At 1 day before AKI, the sensitivity of uNGAL and uATF3 were comparable but uATF3 showed higher specificity.

**Table 2:** Urine neutrophil gelatinase-associated lipocalin (uNGAL) and urine exosomal activating transcriptional factor 3 (uATF3) performances with predetermined cutoff points

Times	Sensitivity	Specificity	LR+	LR-	PPV	NPV
uNGAL						
Cutoff 100 ng/mL						
Baseline	57.1	23.2	0.7	1.9	7.6	83.0
Day-1	98.2	36.4	0.7	1.3	12.1	88.0
Day + 1	90.0	28.1	1.3	0.7	13.8	93.1
Day + 2	100.0	35.4	1.6	0.0	14.7	100.0
Cutoff 150 ng/mL						
Baseline	52.2	34.4	0.8	1.6	9.2	85.2
Day-1	97.5	44.3	1.8	0.2	16.5	96.7
Day + 1	71.4	36.3	1.9	0.5	16.9	95.1
Day + 2	83.3	50.2	1.7	0.3	16.0	96.5
uATF3						
Cutoff 6 ng/mL						
Baseline	57.1	73.2	2.1	0.6	19.1	93.9
Day-1	42.9	73.2	1.6	0.8	15.1	92.0
Day + 1	71.4	80.0	2.3	0.4	20.0	95.6
Day + 2	71.4	90.2	7.3	0.3	44.9	96.6
Cutoff 12 ng/mL						
Baseline	57.1	80.2	5.8	0.5	39.4	95.0
Day-1	93.3	85.4	5.7	0.2	38.8	97.9
Day + 1	90.9	88.2	5.7	0.5	38.2	98.9
Day + 2	84.6	67.5	4.7	0.5	37.8	95.5

*LR*, likelihood ratio; *PPV*, positive predictive value; *NPV*, negative predictive value

Day-1 = the day before AKI was diagnosed, Day + 1 = the day after AKI was diagnosed, Day + 2 = the second day after AKI was diagnosed

In a multiple logistic regression model, patients with uNGAL at 150 ng/mL had a fivefold risk of AKI (OR, 5.6; 95% CI, 3.1 to 7.4); those patients with uATF3 at 12 ng/mL showed 8-fold higher risk of developing AKI (OR, 8.2; 95% CI, 5.6 to 12.8) (Table 3). Presence of comorbidity was associated with an OR of 2.8 (95% CI, 1.6 to 3.8), and high APACHE score were associated with an OR of 4.2 (95% CI, 1.9 to 6.6). Regarding the risk prediction model, a fair performance was obtained (Hosmer–Lemeshow *P* value =0.77), with AUC = 0.79 (95% CI, 0.70 to 0.88). To determine the addition values of uNGAL and uATF3 to the clinical model (age, comorbidity and APACHE II score), the category-free NRI (cfNRI) was calculated (Table 4). Urine NGAL, uATF3, and both values in combination enhanced predictive risk in 67%, 69% and 49%, respectively, in sepsis-AKI patients, and 35%, 44% and 52%, respectively, in sepsis-non-AKI patients. In addition, uNGAL, uATF3, and the combined biomarkers improved overall cfNRI in 75%, 83% and 80%, respectively (Table 4).

**Table 3:** Multiple logistic regression model

Variables	OR	CI	*p*-value
uNGAL	5.6	3.1 to 7.4	<0.001
uATF3	8.2	5.6 to 12.8	<0.001
Age (yr)	1.2	1.0 to 1.3	0.06
Comorbidity	2.8	1.6 to 3.8	0.004
APACHE II	4.2	1.9 to 6.6	<0.001

uNGAL at day + 1 at 150 ng/mL and uATF3 at day + 1 at 12 ng/mL were analyzed with age, comorbidity and APACHE score*u*; *ATF3*, urinary activating transcriptional factor 3; *uNGAL*, urinary neutrophil gelatinase-associated lipocalin; *OR*, odds ratio;*CI*, 95% confidence interval

**Table 4:** Net classification for model improvement with urine neutrophil gelatinase-associated lipocalin (uNGAL) and urine exosomal activating transcriptional factor 3 (uATF3)

Sepsis-AKI	+ uNGAL	+ uATF3	+ uNGAL + uATF3
Goodness-of-fit reference	0.69	0.83	0.88
Goodness-of-fit reference + biomarker (s)	0.75	0.86	0.84
AUROC of reference	0.61 (0.55 – 0.63)	0.74 (0.71-0.78)	0.84 (0.80 – 0.88)
AUROC of reference + biomarker (s)	0.64 (0.54-0.74)	0.84 (0.77-0.91)	0.85 (0.81 – 0.90)
*P*-value (AUC difference)	=0.03	<0.0001	=0.11
cfNRI [events; % (CI)]	47.1 (33.9 – 69.8)	69.3 (66.4 – 82.8)	49.2 (25.5 – 56.8)
cfNRI [non-event; % (CI)]	34.8 (25.4 – 52.3)	44.4 (30.8 – 51.0)	51.8 (43.4 – 58.9)
cfNRI [% (CI)]	74.9 (63.7 – 88.9)	82.5 (74.8 – 90.1)	79.7 (70.8 – 88.8)

The reference is the multiple regression model with age, comorbidity and APACHE II score with uNGAL and uATF3. The calibration of the model was performed by using Hosmer–Lemeshow goodness-of-fit test. AUROC, area under the receiver operating characteristic curve; cfNRI, category-free net reclassification improvement; CI, 95% confidence interval; uATF3, urinary activating transcriptional factor 3; uNGAL, urinary neutrophil gelatinase-associated lipocalin

## Discussion

Urinary exosome is an interesting source of urine biomarker and urinary exosomal ATF3 (uATF3) was a candidate of early AKI biomarker [20, 21]. ATF3 and NGAL expressed in kidney histology earlier than Scr of sepsis mice. Indeed, the analysis of urine patients with sepsis-AKI demonstrated higher uNGAL and uATF3 in comparison with either healthy volunteer or non-sepsis AKI. Moreover, only uATF3, but not uNGAL, increased during the early period of sepsis-AKI with the better area under receiver operating characteristic curve (AUROC) over uNGAL.

To see if ATF3 is a good candidate for the early sepsis-AKI biomarker, we explore in a mouse model due to the known sepsis onset in the model but not in patients. Because sNGAL increased as early as 6 h after cecal ligation and puncture (CLP) with the onset of clinical abnormality consistent with previous publications [10, 27, 31], we determined 6 h after CLP as the early phase of sepsis-AKI. Interestingly, both NGAL and ATF3 expressed in renal tubular cell at brush border and nuclei, respectively, suggested different functions of these proteins. Indeed, NGAL produced and excreted out from different cells in response to organs injury to controlling the extracellular iron reactive oxygen species then re-absorbed and secreted through the proximal and distal renal tubular cell, respectively [32]. On the other hand, ATF3 interfered with several intra-nuclei transcriptional factors to inhibit pro-inflammatory cytokine translational processes [22]. Moreover, activated caspase-3 was also detectable from renal tissue at 6 h of CLP by Western blot analysis support the association of apoptosis and ATF3 or NGAL as previously described [33]. Despite ATF3 is not specific to kidney as it was also demonstrated in other organs as previously known [34–36], it seems to be activated only in condition with organs injury as there was no ATF3 expression in sham mice. Then ATF3 in urine should be a better representative renal biomarker than serum ATF3. NGAL, on the other hand, is also not specific to kidney and kidney injury [37] but it could be used as an early-AKI biomarker [14, 38, 39]. Although, the early expression of NGAL and ATF3 in kidney of CLP mice suggested the possibility of early sepsis biomarker, CLP model was anuria. Then we test uNGAL, a more specific renal injury than plasma NGAL, and exosomal uATF3 in urine patient sample.

Indeed, uATF3 and uNGAL were high in patients with sepsis-AKI in comparison with the healthy volunteers but with the poor correlation between both parameters. The correlation is better with the higher Scr. Perhaps, there were different molecular induction pathways of both molecules in the different sepsis-AKI severity. Interestingly, uATF3 was negative in all healthy volunteers implied the high specificity to sepsis-AKI.

For exploration of early sepsis-AKI biomarkers, we collected urine samples of patients with sepsis during the first week of the admission to identify the onset of sepsis-AKI. To show the tendency of biomarkers toward sepsis-AKI, matched data of uNGAL and uATF3 from all time-point were combined. As expected, uATF3 and uNGAL in sepsis-AKI were higher than sepsis-non-AKI group and uATF3 was rarely increased in sepsis-non-AKI. In contrast, there was a baseline level and slightly increase of uNGAL in healthy volunteers and sepsis-non-AKI during the follow-up. This implied an easier decision for the cut-off value of uATF3 than uNGAL. For the clinical application, uNGAL and uATF3 were analyzed in comparison with the day of increased Scr due to the difference onset of sepsis-AKI in these patients. Surprisingly. uNGAL show a tendency to increase at 1 day before increase in Scr, but non-statistically significance, perhaps due to the non-specific response to infection. The loss of the AKI prediction property of uNGAL in our study might due to the very subtle increased in Scr or the non-oliguric septic-AKI in most of the patients. However, our data derived from the very strict criteria of new onset of sepsis-AKI developed during the admission. Nevertheless, only uATF3 increased at the same days of increased Scr. This demonstrated the benefit of uAFT3 over uNGAL in predicting AKI. Despite uATF3 did not increase before Scr, uATF3 but not uNGAL, would be a good additive biomarker for supporting the onset of AKI in sepsis condition with only a subtle increase of Scr. Further studies are needed.

With all matching data of uATF3 and uNGAL with the mean values of sepsis-non AKI group as the cut-off, the sensitivity of uATF3 for sepsis-AKI diagnosis was comparable with uNGAL with the higher specificity (Table 3). Indeed, our data demonstrated the comparable AUROC of uNGAL and proposed uATF3 as another early sepsis-AKI biomarker (Table 5).

**Table 5:** Summary of the studies in urine and plasma biomarkers for sepsis-related acute kidney injury

Authors (year)	Biomarkers	AUROC	95% CI	Timing of measurement	Threshold values	Sensitivity	Specificity
Urine							
Martensson et al. (2010) [12]	NGAL^a^ (ng/mg creatinine)	0.86	0.68-1.0	12 h following septic shock	>68	0.71	1.0
Su et al. (2011) [40]	sTREM-1^a^ (pg/mL)	0.92	0.85-0.99	48 h before AKI diagnosis^c^	69.04	0.94	0.76
Aydogdu et al. (2013) [41]	Cys-C^a^ (mg/L)	0.86	-	Within 8 days after admission	0.106	0.85	0.80
	NGAL^a^ (ng/mL)	0.80	-		29.5	0.88	0.73
Fan et al. (2014) [30]	NGAL^a^ (ng/mL)	0.86	0.81-0.93	7 days after onset of sepsis	402	0.89	0.74
Matsa et al. (2014) [42]	NGAL^a^ (ng/mL)	0.78	-	24 h after admission	350	0.75	0.82
Terzi et al. (2014) [43]	α1m^a^ (mg/L)	0.74	-	24 h before AKI onset	47.9	0.88	0.62
Dai et al. (2015) [44]	Cys-C^a^ (mg/L)	0.74	0.64-0.84	24 h before AKI onset	N/A	N/A	N/A
	NGAL^a^ (ng/mL)	0.88	0.79-0.95		N/A	N/A	N/A
	sTREM-1^a^ (pg/mL)	0.78	0.69-0.87		N/A	N/A	N/A
The present study	ATF3^b^ (ng/mL)	0.84	0.77-0.91	24 h before AKI onset	12	0.93	0.85
	NGAL^b^ (ng/mL)	0.64	0.54-0.74		150	0.98	0.44
Plasma							
Martensson et al. (2010) [12]	NGAL (ng/mL)	0.67	0.39-0.94	12 h following septic shock	>120	0.83	0.50
Aydogdu et al. (2013) [41]	Cys-C (mg/L)	0.82	-	Within 8 days of admission	1.5	0.73	0.68
	NGAL (ng/mL)	0.44	-		N/A	N/A	N/A
Matsa et al. (2014) [42]	NGAL (ng/mL)	0.88	-	24 h after admission	400	0.79	0.75
Nakamura et al. (2014) [45]	Presepsin (pg/mL)	0.70	-	Within 24 h of admission	670	0.70	0.81
Nakamura et al. (2015) [46]	Procalcitonin (ng/mL)	0.88	-	Within 24 h of admission	0.42	0.95	0.65
Dai et al. (2015) [44]	Cys-C (mg/L)	0.74	0.63-0.84	24 h before AKI onset	N/A	N/A	N/A
	NGAL (ng/mL)	0.83	0.74-0.92		N/A	N/A	N/A
	sTREM-1 (pg/mL)	0.75	0.65-0.85		N/A	N/A	N/A

*α1m*, alpha-1-microglobulin; *AKI*, acute kidney injury; *ATF3*, activating transcriptional factor 3; *AUROC*, area under the receiver operating characteristic curve; *Cys-C*, cystatin-C; *N/A*, data not available; *NGAL*, neutrophil gelatinase-associated lipocalin; *sTREM-1*, soluble triggering receptor expressed on myeloid cells-1

^a^detection from urinary soluble fraction part

^b^detection from urinary exosomal part

^c^no data were available 24 h before AKI onset

In short, our results were a proof of concept that urinary exosome was an interesting source of biomarkers and urinary exosomal ATF3, in particular, was an interesting sepsis-AKI biomarker. More studies will be needed for the proper clinical use of uATF3.

There are several limitations to this study. First, the exosome extraction and analysis method was too complicated for a routine biomarker. The requirement of high urine volume, the normalization by urine creatinine and the Western blot analysis was in-convenient. Second, there were only 14% patients with oliguric sepsis-AKI in our data. Therefore, the levels of uNGAL and uATF3 in oliguric sepsis-AKI might be different. Third, although it was a prospective, cohort study; the number of patients was too small. However, our results could be sufficient to conclude that ATF3 in urine exosome was an interesting sepsis-AKI biomarker that should be supported in the larger studies. Finally, the observation was only a cross-sectional and a short term follow-up data so the association of these biomarkers and clinical outcomes was not explored.

## Conclusion

This study identifies ATF3, a transcriptional factor; in urine exosome was an interesting sepsis-AKI biomarker. Since uATF3 could not be detected in most of the patients with sepsis-non-AKI, the qualitative test of uATF3 might be adequate for detecting sepsis-AKI. In contrast, with the baseline of uNGAL, the debates on the cut-off values of uNGAL for sepsis-AKI are still on-going. Our results were also a proof of concept that urine exosomes were an interesting source of urine biomarkers.

## Abbreviations

AKI: Acute kidney injury
AKIN: Acute Kidney Injury Network
APACHE II: Acute Physiology and Chronic Health Evaluation II
ATF3: Activating transcription factor 3
AUROC: Area under the receiver operating characteristic
cfNRI: Category-free NRI
CKD: Chronic kidney disease
CLP: Cecal ligation and puncture
KDIGO: Kidney Disease Improving Global Outcomes
NGAL: Neutrophil gelatinase-associated lipocalin
Scr: Serum creatinine
sepsis-AKI: Sepsis-induced acute kidney injury
SIRS: Systemic inflammatory response syndrome
uATF3: Urinary exosomal activating transcriptional factor 3
Ucr: Urine creatinine
uNGAL: Urinay neutrophil gelatinase-associated lipocalin
